# Supplementary material for: The Use of Infographics to Inform Infection Prevention and Control Nursing Practice: A Descriptive Qualitative Study
Source: Healthcare (Basel). 2025 Nov 18;13(22):2961. doi: 10.3390/healthcare13222961 (PMC12652311; doi:10.3390/healthcare13222961)
Supplement: Supplementary file 1 [file healthcare-13-02961-s001.zip › Tabel S2 - COREQ Checklist.pdf]

healthcare

*Article*

# The Use of Infographics to Inform Infection Prevention and Control Nursing Practice: A Descriptive Qualitative Study

**Susana Filipe [1,2,*], Maria Manuel Borges [3], Amélia Castilho [1] and Celeste Bastos [4]**

1 Health Sciences Research Unit: Nursing (UICISA: E), Nursing School of Coimbra (ESEnfC), Coimbra, Portugal
2 Local Health Unit of Baixo Mondego, Figueira da Foz, Portugal; susanafilipe@ulsbm.min-saude.pt
3 Univ. Coimbra, CEIS20, Coimbra, Portugal
4 CINTESIS@RISE, Nursing School of Porto (ESEP), Porto, Portugal
* Correspondence: susanafilipe@ulsbm.min-saude.pt

## COREQ: Consolidated criteria for reporting qualitative research: a 32-item checklist for interviews and focus groups

| Section/Topic | Item No | Checklist item | Reported on page No |
|---|---|---|---|
| **Domain 1: Research team and reflexivity** | | | |
| Personal Characteristics | | | |
| *Interviewer/facilitator* | 1 | Which author/s conducted the interview or focus group? Interviewer/facilitator | The interviews were conducted by the first author (acknowledged on page 4) |
| *Credentials* | 2 | What were the researcher's credentials? E.g. PhD, MD | The first author is a PhD student a RN and MSc (these credentials are acknowledged on page 1 – ORCID 0000-0001-8758-8123) |
| *Occupation* | 3 | What was their occupation at the time of the study? | The first author is the Institution's IPC Lead Nurse (acknowledged on page 15) |
| *Gender* | 4 | Was the researcher male or female? | Female gender |
| *Experience and training* | 5 | What experience or training did the researcher have? Relationship with participants | The first author had received formal training in qualitative interviewing and analysis as part of her PhD programme, and her tutors (senior qualitative researchers) provided guidance in designing the interview guide, checking transcripts, and refining coding and theme development.<br>The researcher had no direct hierarquical relation with the participants, although, as the Institution's IPC lead nurse she has a close professional relationship with the IPC link nurses (acknowledged on pages 4 and 15) |
| Relationship with participants | | | |
| *Relationship established* | 6 | Was a relationship established prior to study commencement? | The first author is the Institution's IPC lead nurse, and before the study she has *worked with* the participants in meetings and operational tasks (acknowledged on page 15) |
| *Participant knowledge of the interviewer* | 7 | What did the participants know about the researcher? e.g. personal goals, reasons for doing the research | The participants were informed of the aims and context of the study on Informed Consent (acknowledged on page 3) |
| *Interviewer characteristics* | 8 | What characteristics were reported about the interviewer/facilitator? e.g. Bias, assumptions, reasons and interests in the research topic | The first author held regular review sessions with her tutors to discuss decisions and emerging themes, thereby identifying and bracketing her assumptions and promoting analytic transparency (on page 15) |

healthcare

MDPI

# COREQ: Consolidated criteria for reporting qualitative research: a 32-item checklist for interviews and focus groups (cont.)

| Section/Topic | Item No | Checklist item | Reported on page No |
|---|---|---|---|
| **Domain 2: study design** | | | |
| Theoretical framework | | | |
| *Methodological orientation and Theory* | 9 | What methodological orientation was stated to underpin the study? e.g. grounded theory, discourse analysis, ethnography, phenomenology, content analysis | The methodological orientation underpinning the study was content analysis (page 4) |
| Participant selection | | | |
| *Sampling* | 10 | How were participants selected? e.g. purposive, convenience, consecutive, snowball | Participants were purposively selected because of their dual role as intermediaries between clinical teams and the IPC team (acknowledged on page 2) |
| *Method of approach* | 11 | How were participants approached? e.g. face-to-face, telephone, mail, email | Participants were approached face-to-face and invited to take part in the study (acknowledged on page 3) |
| *Sample size* | 12 | How many participants were in the study? | 13 participants were included in the study (page 4) |
| *Non-participation* | 13 | How many people refused to participate or dropped out? Reasons? | All those approached agreed to participate; there were no refusals or drop-outs (page 3) |
| *Setting of data collection* | 14 | Where was the data collected? e.g. home, clinic, workplace | Data were collected at workplace (in a private room meeting) for face-to-face interviews, and via zoom® for two interviews. Acknowledged on page 4) |
| *Presence of non-participants* | 15 | Was anyone else present besides the participants and researchers? | No one else besides the participant and the interviewer was present during the sessions |
| *Description of sample* | 16 | What are the important characteristics of the sample? e.g. demographic data, date | Participants were eligible if they served as IPC link nurses in the hospital setting (acknowledged on page 3) |
| Data collection | | | |
| *Interview guide* | 17 | Were questions, prompts, guides provided by the authors? Was it pilot tested? | The interview guide was provided, but it hasn't been pilot tested (acknowledged on page 4) |
| *Repeat interviews* | 18 | Were repeat interviews carried out? If yes, how many? | There was no need to repeat interviews |
| *Audio/visual recording* | 19 | Did the research use audio or visual recording to collect the data? | The researcher used audio recording to collect the data (acknowledged on page 4) |
| *Field notes* | 20 | Were field notes made during and/or after the interview or focus group? | There were no field notes taken |
| *Duration* | 21 | What was the duration of the interviews or focus group? | Interviews lasted between 40 and 75 minutes (acknowledged on page 4) |
| *Data saturation* | 22 | Was data saturation discussed? | After the 10th interview, no new themes emerged, and this was discussed with the research team (acknowledged on page 4) |
| *Transcripts returned* | 23 | Were transcripts returned to participants for comment and/or correction? | Transcripts were returned to participants for comments, and no corrections were asked for (acknowledged on page 4) |

healthcare

MDPI

# COREQ: Consolidated criteria for reporting qualitative research: a 32-item checklist for interviews and focus groups (cont.)

| Section/Topic | Item No | Checklist item | Reported on page No |
|---|---|---|---|
| Domain 3: analysis and findings<br>Data analysis | | | |
| *Number of data coders* | 24 | How many data coders coded the data? | Data were coded by the research team (acknowledge on page 5) |
| *Description of the coding tree* | 25 | Did authors provide a description of the coding tree? | The authors provided a description of the coding tree on page 5 |
| *Derivation of themes* | 26 | Were themes identified in advance or derived from the data? | Themes were derived from the data, as acknowledged on page 4 |
| *Software* | 27 | What software, if applicable, was used to manage the data? | Data were analyzed using AIQDA® software, enabling segmentation of verbatim statements and facilitating the coding process (acknowledged on page 4) |
| *Participant checking* | 28 | Did participants provide feedback on the findings? | Participant checking was offered but none provided feedback |
| Reporting | | | |
| *Quotations presented* | 29 | Were participant quotations presented to illustrate the themes / findings? Was each quotation identified? e.g. participant number | Participant quotations were presented and identified with a code in the Results section, to illustrate the themes (on pages 6 to 11) |
| *Data and findings consistent* | 30 | Was there consistency between the data presented and the findings? | Findings are grounded in the data, nonetheless, we demonstrate consistency by providing quotes that are linked to the themes and show how interpretations follow from data (on pages 5 to 11) |
| *Clarity of major themes* | 31 | Were major themes clearly presented in the findings? | The findings were organized in the Results section, which was structured from the Themes and Categories (on pages 5 to 11) |
| *Clarity of minor themes* | 32 | Is there a description of diverse cases or discussion of minor themes? | The authors also presented minor themes, or sub-categories in the results section (on pages 5 to 11) |
