# Supplementary material for: The Use of Infographics to Inform Infection Prevention and Control Nursing Practice: A Descriptive Qualitative Study
Source: Healthcare (Basel). 2025 Nov 18;13(22):2961. doi: 10.3390/healthcare13222961 (PMC12652311; doi:10.3390/healthcare13222961)
Supplement: Supplementary file 1 [file healthcare-13-02961-s001.zip › Table S1 - Interview guide.pdf]

Title: Using infographics as a catalyst to improve infection prevention and control practice: a case study with nurse link practitioners

TOPIC	OBJECTIVE	QUESTIONS
A. Legitimize the interview	A1. To explain the context and purpose of the interview<br>A2. Ensure confidentiality<br>A3. Obtain permission to record the interview	A1. Inform the link practitioner about the research and it's purpose<br>A2.Explain how the interview will be used, how his participation is important to this project's success. (If the link practitioner is happy for his perspective to be used in that way, follow the interview. Otherwise, the interview won't continue)<br>A3. Explain taht confidentiality will be ensured, that is, their name won't be attached to the information or will be made public, unless they ask for this to happen<br>A4. Ask for permission to record the interview<br>A5. Thank for the cooperation
B. Characterization	B1. Gender, age<br>B2. Years of practice<br>B3. Years as link practitioner	B1. Inform that data about gender, age, years of practice and as a link practitioner only serve the purpose of characterization
C. Healthcare Associated Infection's (HAI's) surveillance data dissemination	C1. Identify how the link practitioners are aware of HAI's surveillance data<br>C2. Understand how the link practitioners feel about the HAI's surveillance dissemination channels	Each year the Infection Prevention and Control (IPC) team issues a report presenting the HAI's surveillance data, where microorganism's patterns and HAI's are analysed.<br>C1. Are the nurses in your ward aware of this data?<br>C2. And what about you? Are you aware of this? How did you become aware of this report?<br>C3. How do you feel about the way the IPC team disseminates the HAI's surveillance data?<br>C4. In your opinion, how could the IPC team improve HAI's surveillance data dissemination or feedback?
D. HAI's surveillance data given relevance	D1. Understand the importance that link practitioners attach to HAI's surveillance data	One of the core components for the implementation of an infection prevention and control program is the HAI's surveillance.<br>D1. In your opinion, why are HAI's surveillance data important?<br>D2. In what way do you believe that being aware of this data can improve practice?<br>D3. Could you give some practical examples on how you believe this to be possible? (practice improvement)
E. The use of infographics to HAI's surveillance dissemination	E1. Understand how the link practitioners relate to data presented in the infographic<br>E2. Understand the potential for the link practitioners to relate with the HAI's surveillance data presented in infographic<br>E3. Identify relevant information to be presented in the infographic	Last year (2022), for the first time, the IPC team issued an infographic that summarized the HAI's surveillance data.<br>E1. How do you feel about the way data are presented in an infographic? (If the respondent doesn't elaborate - Is it easier to understand, more intuitive or does it allow to visualize data as a whole?)<br>E2. What did the infographics tell you about HAI data?<br>E3. Did the infographics give you new insights?<br>E4. In your opinion, what are the advantages of this type of feedback? And the disadvantages?<br>E5. In your opinion, can the use of infographics be a catalyst to, or encourage, improved infection prevention and control practices?<br>E6. In your opinion, what is the most relevant information to be addressed in HAI's surveillance infographic?
